# COVID-19 Case Recognition from Chest CT Images by Deep Learning, Entropy-Controlled Firefly Optimization, and Parallel Feature Fusion

**DOI:** 10.3390/s21217286

**Published:** 2021-11-02

**Authors:** Muhammad Attique Khan, Majed Alhaisoni, Usman Tariq, Nazar Hussain, Abdul Majid, Robertas Damaševičius, Rytis Maskeliūnas

**Affiliations:** 1 Department of Computer Science, HITEC University, Taxila 47080, Pakistan; attique.khan@hitecuni.edu.pk (M.A.K.); nazar.hussain@hitecuni.edu.pk (N.H.); abdul.majid@hitecuni.edu.pk (A.M.); 2 College of Computer Science and Engineering, University of Ha’il, Ha’il 55211, Saudi Arabia; majed.alhaisoni@gmail.com; 3 Information Systems Department, College of Computer Engineering and Sciences, Prince Sattam Bin Abdulaziz University, Al Khraj 11942, Saudi Arabia; u.tariq@psau.edu.sa; 4 Faculty of Applied Mathematics, Silesian University of Technology, 44-100 Gliwice, Poland; 5 Department of Multimedia Engineering, Kaunas University of Technology, 51368 Kaunas, Lithuania; rytis.maskeliunas@ktu.lt

**Keywords:** COVID-19, deep learning, feature fusion, firefly algorithm, medical imaging

## Abstract

In healthcare, a multitude of data is collected from medical sensors and devices, such as X-ray machines, magnetic resonance imaging, computed tomography (CT), and so on, that can be analyzed by artificial intelligence methods for early diagnosis of diseases. Recently, the outbreak of the COVID-19 disease caused many deaths. Computer vision researchers support medical doctors by employing deep learning techniques on medical images to diagnose COVID-19 patients. Various methods were proposed for COVID-19 case classification. A new automated technique is proposed using parallel fusion and optimization of deep learning models. The proposed technique starts with a contrast enhancement using a combination of top-hat and Wiener filters. Two pre-trained deep learning models (AlexNet and VGG16) are employed and fine-tuned according to target classes (COVID-19 and healthy). Features are extracted and fused using a parallel fusion approach—parallel positive correlation. Optimal features are selected using the entropy-controlled firefly optimization method. The selected features are classified using machine learning classifiers such as multiclass support vector machine (MC-SVM). Experiments were carried out using the Radiopaedia database and achieved an accuracy of 98%. Moreover, a detailed analysis is conducted and shows the improved performance of the proposed scheme.

## 1. Introduction

At the end of 2019, a new illness originated from a coronavirus appeared in the Hubei province of China and rapidly spread worldwide in 2020 [1]. This disease was named COVID-19 by the World Health Organization (WHO) in February 2020 [2]. COVID-19 disease is caused by the virus named SARS-CoV-2 [3]. This disease may cause organ failure and respiratory difficulties in severe cases [4]. In addition to the medical impact, the disease had a significant effect on the global economy and the environment [5].

The typical reverse transcription polymerase chain reaction (RT-PCR) test is a tedious procedure to recognize COVID-19 [6]. Artificial intelligence (AI) techniques have been deployed to combat the epidemic caused by COVID-19 and its negative consequences [7], and, specifically, for medical diagnostics [8]. Utilizing deep learning (DL), a modern form of machine learning, this disease can be detected and identified at early stages from the X-ray and CT frames of the chest [9,10,11]. The most common diagnostic X-ray examination is the chest X-ray. Using a tiny burst of radiation that travels through the body, a chest X-ray machine can generate pictures of the lungs and chest. Radiation creates a picture on a photographic film or a specific sensor.

CT is the most sensitive imaging modality for detecting pulmonary problems. The images are captured using a CT scanner, which takes multiple cross-sectional slice images of the patient’s body in succession. The sensor array is a critical component of a contemporary multi-slice CT scanner; it detects X-ray energy that has been partially attenuated by body tissues and transforms it into a digital output. This digital signal contains all the information needed by the image processor, which reconstructs slices from continuous projections of X-ray image data recorded at various rotation angles of the CT equipment, and then displays them as 3D volumes of the patient’s body parts. Such devices and sensor systems are increasingly being used as a part of the Internet of Medical Things (IoMT) that connect to healthcare information systems through online computer networks.

Convolutional neural network (CNN) models have shown their importance in the development of automated detection systems for COVID-19 pneumonia [12,13]. Several techniques [14,15] have been introduced to identify COVID-19, which utilized the deep CNN features and produced more accurate results than handcrafted features-based systems [16]. A deep CNN model COVIDNet-CT [17] was introduced to diagnose and recognize COVID-19 in chest CT frames. In [18], researchers introduced the generative adversarial network (GAN) with pre-trained AlexNet, ResNet18, and GoogleNet models to recognize coronavirus infection in chest X-ray images. This method achieves promising results on the GoogleNet model. Other DL-based methods for the detection of the spread of COVID-19 infection include [19,20] and many more [21,22]. In the pandemic breakout, IoT can detect, track, and isolate COVID-19 patients more efficiently. The use of IoT devices to detect and recognize COVID-19 can decrease the detection time and help detect the disease. Some researchers used IoT technology to detect COVID-infected persons [23,24]. The IoT models help to isolate the infected one by monitoring body temperature.

Recently, many techniques have been presented to recognize and identify COVID-19 in X-ray images [25,26,27] and CT frames [28,29]. These methods use the different deep CNN features to develop a more accurate detection model [30,31,32]. In [14], researchers calculate the classification results using ten famous CNN models. These models classify CT images collected from 108 patients with COVID-19. They concluded that, after extensive experiments, ResNet-101 produced more accurate results with respect to sensitivity and accuracy. This model achieved 100% sensitivity and 99.51% accuracy. Ko et al. [33] presented a framework for recognizing COVID-19 based on a single CT frame. This 2D deep learning framework was developed using the transfer learning technique. The name of this model is the fast-track COVID-19 classification network (FCONet). They performed experiments on four deep CNN models, including Inception-V3, VGG16, Xception, and ResNet-50. In the FCONet framework, the highest results were achieved with ResNet-50. The calculated specificity and recognition rates are 100% and 99.87%, respectively.

A weakly supervised deep learning model [34] was proposed for the recognition of COVID-19 infection. This model helped reduce the manual labeling requirements of CT images. Gao et al. [35] introduced a dual branch combination network (DCN) system to segment and classify the lesion in COVID-19-infected CT frames. They performed experiments on a privately collected dataset from 1202 patients. This DCN model achieved 96.74% classification accuracy. In [36], the proposed methodology was used to detect and segment COVID-19 infection in CT frames. The results of this model were compared with two radiology experts and performed much faster. Horry et al. [37] used ultrasound, X-ray, and CT images to detect COVID-19. They developed their model using transfer learning in the VGG19 model. This proposed model obtained precision of 86%, 100%, and 84% for X-ray, ultrasound, and CT images, respectively. Ozturk et al. [38] proposed an automated detection model for recognizing COVID-19 in chest X-ray frames using binary classification and multiclass classification. In this model, researchers used the DarkNet model-based classifier. They used 17 convolution layers and performed a filtering process on each layer. The proposed model achieved 98.08% accuracy for two classes and 87.02% for multi-classes. In [39], researchers combined CNN with long short term memory (LSTM) to automatically detect the COVID-19 in X-ray frames. This model extracts features from the CNN model, and LSTM is utilized for infection detection from extracted features. The maximum accuracy achieved with this model is 99.4% and an AUC of 99.9%.

In [40], the effectiveness of few-shot learning in U-Net architectures was investigated, which allows for dynamic fine-tuning of the network weights when few new samples are introduced into the U-Net. The results of the experiments show that the accuracy of segmenting COVID-19-infected lung areas has improved. In [41], the X-ray image features were extracted using the histogram-oriented gradient (HOG) and fused with the CNN features to construct the classification model. For enhanced edge retention and image denoising, the modified anisotropic diffusion filtering (MADF) technique was used. The substantial fracture zone in the raw X-ray images was identified using a watershed segmentation approach. With a testing accuracy of 99.49%, specificity of 95.7%, and sensitivity of 93.65%, this ensured a satisfactory performance in terms of recognizing COVID-19. In [42], a novel probabilistic model was created based on a linear combination of Gaussian distributions (LCG). The authors modified the standard expectation-maximization (EM) algorithm to estimate both dominant and subdominant Gaussian components, which are used to refine the final estimated joint density sequentially. In 3D CT scans, the approach was used to segment the COVID-19-affected lung region. In [43], flu symptoms, throat discomfort, immune status, diarrhea, voice type, breathing difficulty, chest pain, and other symptoms were employed to predict the likelihood of COVID-19 infection using machine learning methods, which achieved a prediction accuracy of more than 97%.

An automated system is required to identify the COVID-19 case based on the X-ray images. It is the cheapest method compared with the COVID-19 test (RT-PCR). However, manual inspection of these images is a hectic and time-consuming process. An experienced radiologist is always required for correct identification. Therefore, it is essential to identify these scans using an automated technique as early as possible. Computerized methods help the radiologist in clinics to support their manual result and detect COVID-19.

In this paper, we proposed a fully automated system using the fusion of features from two deep learning networks. Our significant contribution to this work is as follows:

- A hybrid contrast enhancement technique is proposed by sequentially employing linear filters.
- Transfer learning is performed by fine tuning the parameters of two deep CNN models.
- Features are extracted from both models and an entropy-controlled Firefly optimization algorithm is implemented for optimal features’ selection.
- Selected optimal features are fused using a parallel positive correlation approach.

The rest of the manuscript is organized as follows. The proposed methodology (i.e., a technique for contrast enhancement, deep learning features, entropy-controlled Firefly based selection of best features, and fusion) is presented in Section 2. The results are discussed in Section 3. Finally, the conclusion of this technique is given in Section 4.

## 2. Methodology

**Figure 1:**
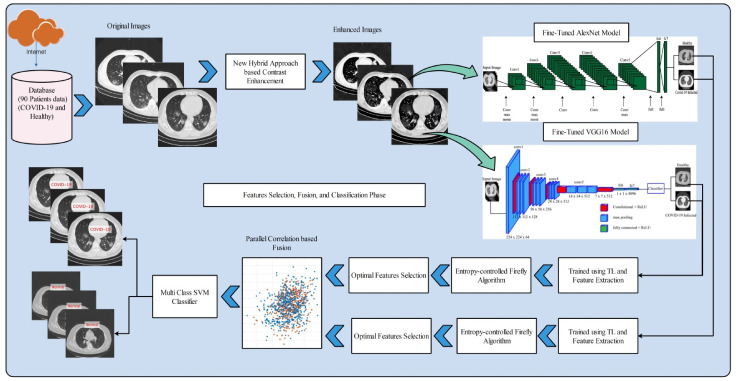
Proposed architecture of X-ray image classification framework using deep learning feature fusion for COVID-19 case recognition.

The proposed COVID-19 classification method using optimal deep learning feature fusion is presented in this section with detailed visual effects and mathematical descriptions. Figure 1 shows the proposed architecture of the COVID-19 classification. This figure explains that, initially, the images are acquired from the Internet and labeled as COVID-19-infected and normal according to the given details. After that, a new hybrid approach is proposed for contrast enhancement. Features are extracted from both models and optimized using a novel entropy-controlled Firefly algorithm. Selected optimal features are fused using a new approach, named parallel positive correlation. Finally, the MC-SVM is used for the classification into normal or COVID-19-infected cases.

### 2.1. Dataset Preparation

**Figure 2:**
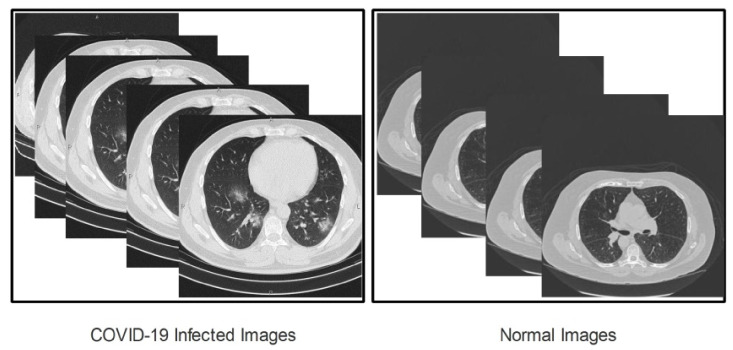
Sample of COVID-19-positive and normal (healthy) images.

The first step in any computerized approach is based on the nature of the database. In this paper, chest CT images of COVID-19-positive and normal images are considered for classification. We collected a total of 2500 COVID-19 images of 90 patients from the Radiopaedia database. On this website, more than 100 chest CT images are available. We consider the images of the first 90 patients for the COVID-19-positive class. We also collected 2000 images from the same website for normal (healthy) patients. All images are in gray scale format. We performed pre-processing and resized the images to a dimension of  512×512. Later, we increase the dataset using the data augmentation process, and the number of images in each class is 6000. In Figure 2, some sample images are illustrated.

### 2.2. Contrast Enhancement

The enhancement of input image contrast is an important and useful step to improve an image’s visual quality [44,45,46]. The primary motivation of this step is to visualize the COVID-19-positive images with more clarity. A hybrid technique is proposed in this paper, based on the combination of two filters: (i) top-hat filtering and (ii) Wiener filter. The output of both filters is passed in a new activation function for final enhancement.

Given ℧ is a database of n images and $℧\in {\mathbb{R}}^{n}$, where each image is represented by $I^{n}\left(x,y\right)$ and $\left(x,y\right)\in \mathbb{R}$. Each image $I^{n}\left(x,y\right)$ has a dimension of N×M and N=M=512. The nature of each image in the database ℧ is grayscale. Consider that e is a structuring element with a value of 21 and ∘ is an opening operator, then top-hat filtering operation is defined as follows:

(1) $$I^{top}\left(x,y\right)=I^{n}\left(x,y\right)-\left(I^{n}\left(x,y\right)\;\circ e\right)$$

The contrast of the image is enhanced using the mentioned filter. Next, the Weiner filter is employed for the removal of noise from image. This filter minimizes the mean square error (MSE) among the estimated random process and the desired process. Mathematically, it is defined as follows:

(2) $$W^{mse}\left(x,y\right)=\frac{W*\left(x,y\right)}{{\left|W\left(x,y\right)\right|}^{2}+\Delta }$$

(3) $$W\left(x,y\right)=e^{-\left(\frac{x^{2}+y^{2}}{2\sigma^{2}}\right)}$$

Here, Δ is a constant, and the value is initialized as 1. The resultant values of $I^{top}\left(x,y\right)$ and $W^{mse}\left(x,y\right)$ are passed in the activation function. The activation function is defined as follows:

(4) $$I^{act}\left(x,y\right)=\left(I^{top}\left(x,y\right)+W^{mse}\left(x,y\right)\right)-I^{n}\left(x,y\right)\times \sigma \left(I^{act}\left(x,y\right)\right)$$

**Figure 3:**
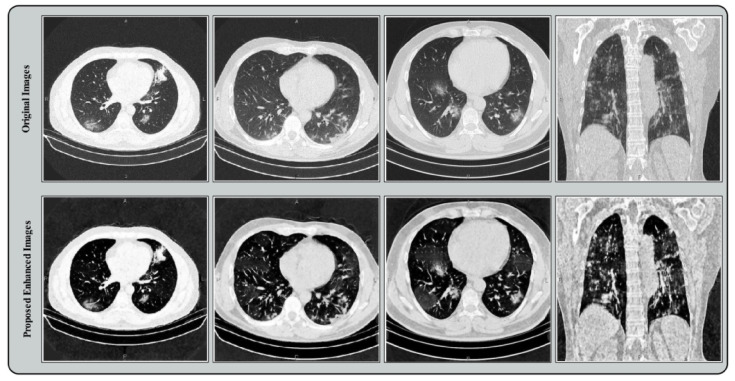
Proposed contrast enhancement effects on the original chest CT images.

The output of this function is presented in Figure 3. The original CT images are illustrated in the first row, and the bottom row shows the intensified images. Based on these resultant images, it can be demonstrated that infected information is visualized with more clarity. These enhanced images are used in the next process for learning a model.

### 2.3. Modified AlexNet Deep Learning Model

To perform computer vision tasks like object detection and classification, AlexNet [47] is a widely used deep convolutional neural network (CNN) capable of attaining higher accuracies on challenging datasets. It has eight depth layers, five convolutional layers, and two fully connected layers with a Softmax layer of 1000 classes. The filter size utilized in convolutional layers is 11×11 and 5×5. Rectified linear units (ReLUs) are used as an activation function owing to their advantage of less computational time. ReLUs are implemented after every convolutional layer. This model was trained on the ImageNet [48] challenging dataset having 1000 object classes. The input size of the CNN model is 227×227×3. The CNN model utilizes regularization to cope with the problem of over fitting. Regularization increased the training time with 0.5 dropouts.

**Figure 4:**
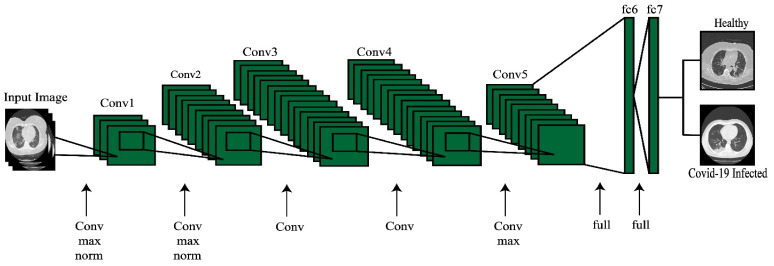
Fine-tuned AlexNet pre-trained model for healthy vs. COVID-19-infected classification.

In this work, we fine-tuned the AlexNet model and eliminate the last layer. A new layer was added, consisting of two target classes: COVID-19 and normal (healthy). The new fine-tuned model was trained through transfer learning (TL) [37], leading to a new modified target model. The modified AlexNet model after the fine-tuning process is shown in Figure 4. The features are extracted from the last layer (FC7) and saved in a new matrix of dimension N×4096, and the mathematically featured matrix is denoted by $\Phi_{N}^{k1}$. Here, k1 denotes the feature vector length and N represents the number of images.

### 2.4. Modified VGG16 Deep Learning Model

The VGG16 [49] convolutional neural network (CNN) is trained on an extensive image database ImageNet [48], having over a million images and 1000 classes. This model achieved 92.7% accuracy on the ImageNet database by securing a top five accuracy position on the ImageNet image recognition challenge. The input size for VGG16 is 224×224×3. This model improved the deficiencies in AlexNet by reducing the filter size on the first and second convolutional layers. The previous filter size was 11×11 and 5×5, which decreased to a 3×3 filter size. The image fed in this model has a size of 224×224. The image passes from multiple convolutional layers having different filter sizes varying from 3×3 to 1×1. The stride is fixed at 1 pixel. The pooling process is performed by deploying five pooling layers and a filter size of 2×2 with a stride of 2. Three fully connected layers after the stack of convolutional layers were added. The first two FC layers have 4096 features. The last fully connected layer expresses the number of classes 1000 of the ImageNet database for which the network was trained.

**Figure 5:**
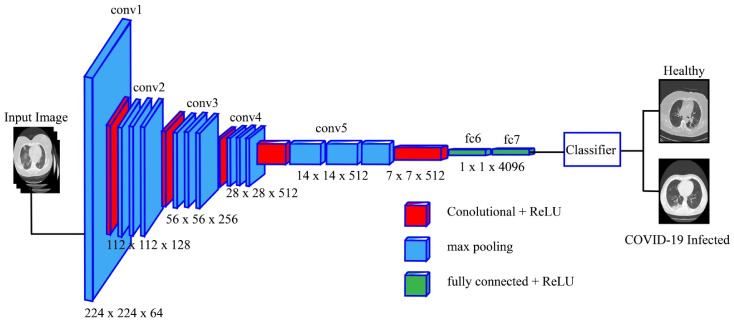
Modified VGG16 deep model for healthy vs. COVID-19-infected image classification.

We fine-tuned this model and removed the last classification layer with a new layer of two output classes: COVID-19 and normal. The fine-tuned model was trained through TL, leading to a new target model. The modified VGG16 model is shown in Figure 5. This target model is now used for feature extraction. Features are extracted from the FC layer seven and receive a resultant feature vector of dimension N×4096, and the mathematically featured matrix is denoted by $\Phi_{N}^{k2}$. Here, k2 denotes the feature vector length, and N represents the number of images.

### 2.5. Feature Selection

In the last decade, feature selection techniques have shown great success in computer vision, particularly in medical imaging, to make the system more efficient [50,51]. In feature selection techniques, the features are not altered like when using feature reduction techniques (such as principal component analysis, PCA) [52]. Subsets of features are selected from the input feature vector for the classification task. This is a primary motivation behind the use of feature selection.

We implemented an entropy-controlled Firefly algorithm (FA) for optimal feature selection. Initially, features are selected through the FA, and later, an entropy-based activation function is proposed and features are passed for the final selection phase. FA is a contemporary and widely used metaheuristic optimization approach, developed by Yang et al. [53], which originated from the glowing conduct of fireflies. Different species of fireflies have a particular flashing sequence. The process of biological luminous produces flashing light. The flashing pattern has two fundamental functions: prey attraction and attraction towards mating partner. FA adopts the flashing behavior of fireflies for the optimization of multimodal problems, and achieved robust performance compared with the particle swarm optimization (PSO) and the genetic algorithm (GA) [54].

Three main steps define FA: (i) A firefly appeals to all other fireflies, and the appeal is not gender-specific. (ii) The magnetism of flies is proportional to their glowing. The glowing fly will attract the fly with low brightness. Greater luminosity leads to a lesser distance between the fireflies. (iii) Lastly, the brightness of fireflies is mapped through a fitness function. The luminosity of a firefly with origin brightness Y is expressed as follows:

(5) $$Y\left(s\right)=Y_{0}e^{-\delta s}$$

where $Y_{0}$ describes the origin of brightness, the distance between two fireflies is expressed as s, and δ is the coefficient of light responsible for luminous intensity and occupation. As we know, brightness and attractiveness are proportional to each other; hence, attraction T can be expressed as follows:

(6) $$T\left(s\right)=T_{0}e^{-\delta s}$$

when s=0, the attractiveness is $T_{0}$. The attraction of Firefly l and m is expressed as follows:

(7) $$P_{l}^{z+1}=T_{0}e^{-\delta s_{lm}^{2}}\left(P_{m}^{z}-P_{l}^{z}\right)+\varphi \left(Rand-0.5\right)$$

where φ describes the parameter randomness, z is the number of iterations, and Rand generates a random number between 0 and 1. The distance between the lth and mth Firefly is denoted by $s_{lm}$ and can be elaborated as follows:

(8) $$s_{lm}=\|P_{m}-P_{l}\|=\sqrt{\sum_{k=1}^{K}{\left(P_{lk}-P_{mk}\right)}^{2}}$$

Based on the distance, the minimum distance features are evaluated. For the evaluation, a MC-SVM classifier was utilized. Based on the error rate, the next iteration is performed. As in this paper, we selected the total iteration number as n=100. After all iterations, an optimal vector was obtained with dimensions of N×1746 and N×1822 for feature vectors $\Phi_{N}^{k1}$ and $\Phi_{N}^{k2}$, respectively. An entropy-based activation function is used for all features for later stage selection. In this stage, features are further refined using the entropy-based activation function. The activation function is defined as follows:

(9) $$H\left(\Phi \right)=-\overset{k}{\underset{i=1}{\sum }}P\left(\Phi^{k}\right)log_{2}P\left(\Phi^{k}\right)$$

(10) $$\tilde{\Phi_{N}^{k1}}=\left\{\forall \Phi_{N}^{k1}\geq H\left(\Phi \right)\right\}$$

(11) $$\tilde{\Phi_{N}^{k2}}=\left\{\forall \Phi_{N}^{k2}\geq H\left(\Phi \right)\right\}$$

where $k\in \left(k1,k2\right)$, $\tilde{\Phi_{N}^{k1}}$ is an optimal selected vector for $\Phi_{N}^{k1}$, and $\tilde{\Phi_{N}^{k2}}$ is an optimal selected vector for $\Phi_{N}^{k2}$, respectively. In this paper, the length of optimal feature vectors after applying the activation function is N×1346 and N×1322, respectively.

The details are explained and given in Algorithm 1.
**Algorithm 1.** FA-Based Feature Optimization.**Start** **Step 1:**$\;\mathrm{Define}\;\mathrm{fitness}\;\mathrm{function}:\;h\left(x\right),\;x=\left(x_{1},\;x_{2},\;\ldots ,\;x_{d}\right)$ **Step 2:**$\;\mathrm{Generate}\;\mathrm{initial}\;\mathrm{population}\;\mathrm{of}\;\mathrm{fireflies}\;P_{l}$, where l=1, 2, 3, …, n **Step 3:**$\;\mathrm{Compute}\;\mathrm{Brightness}\;Y\left(s\right)=Y_{0}e^{-\delta s}$ **Step 4:** Define Absorption Coefficient δ  - While(z<MaxGeneration)    - for l=1:𝕟      - for m=1:l        - $if\;(Y_{m}>Y_{l})$        - Vary attractiveness with distance s via $e^{-\delta s}$        - Move firefly l towards m using $P_{l}^{z+1}=T_{0}e^{-\delta s_{lm}^{2}}\left(P_{m}^{z}-P_{l}^{z}\right)+\varphi \left(Rand-0.5\right)$        - Evaluate new solutions and update brightness        - end if      - end for m    - end for l    - Find the latest best Firefly    - Entropy-based activation is applied $H\left(\Phi \right)$    - Best Optimal Features are Selected $\tilde{\Phi_{N}^{k}}$  - end While  - Processing results and visualization **End**

### 2.6. Feature Fusion and Classification

Feature fusion is an important method in pattern recognition [55]. It is used to combine or aggregate features originating from multiple inputs such as different types of images, different feature generation methods, or different layers of trained deep learning models [56,57]. Feature fusion is an important step in the proposed methodology, in which we fuse the information of both selected optimal deep feature vectors.

In this paper, we propose a new fusion approach, named parallel positive correlation. Initially, both vectors’ lengths were equalized according to the size of the maximum length vector. As the length of $\tilde{\Phi_{N}^{k1}}$ is higher than vector $\tilde{\Phi_{N}^{k2}}$, we performed zero padding. Based on the zero padding, we made the length of both vectors equal and then determined the correlation between the pair of features as i and j. The positively correlated features are selected for each i and j. The positive correlation denotes the features that have a correlation value close to one.

In the output, a vector size of dimension N×1346 was obtained for the final classification. The multiclass SVM (MCSVM) [58] was utilized as a classifier for final feature classification.

## 3. Results and Analysis

For the experiment, we collected 90 patients’ data. Half of images are used to train a model, while the other half of the images are selected for the testing results. Tenfold cross-validation is performed for all the results. The other deep learning parameters of learning rate, mini batch size, number of epochs, and learning method are 0.001, 64, 200, and stochastic gradient descent, respectively. Multiple classifiers are utilized in the experiments, including naïve Bayes, fine tree, ensemble learning, and decision trees. Each classifier’s performance is computed through several measures: sensitivity rate, precision rate, F1-score, accuracy, and false negative rate (FNR). Moreover, the computational time is also calculated to analyze the proposed method in the real-time testing phase.

All the simulations are conducted in MATLAB2020b (MathWorks Inc., Natick, MA, USA) using a desktop computer with Intel Core i7 of 512 SSD and 32 GB RAM and a 16 GB GPU.

### 3.1. Results

**Figure 6:**
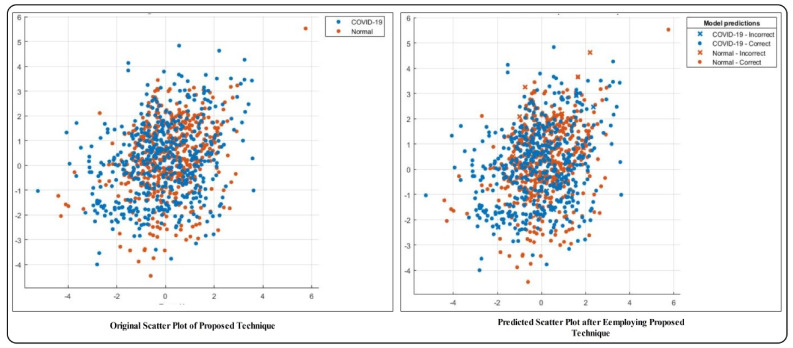
Scatter plot of true classes and predicted classes by MC-SVM using the proposed technique.

**Figure 7:**
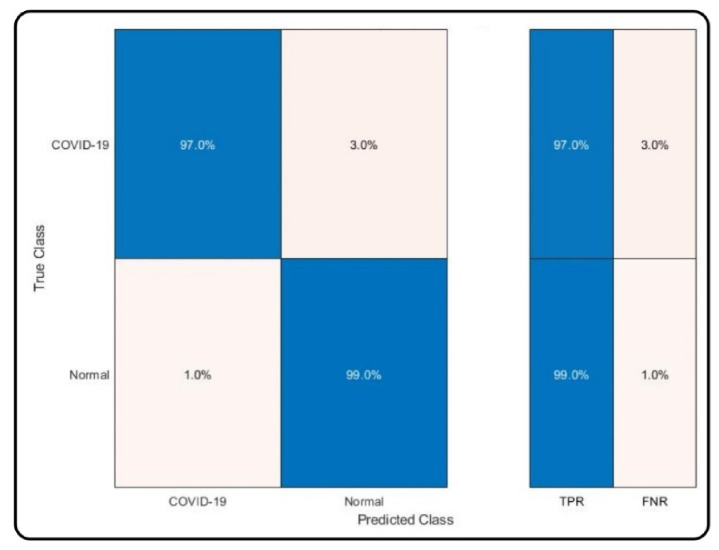
Confusion matrix of the MC-SVM classifier using the proposed technique.

The results of the proposed method for several classifiers including MC-SVM, DT (decision tree), LDA (linear discriminant analysis), KNB (kernel naïve bayes), QSVM (quadratic SVM), F-KNN, cosine KNN, and EBT (ensemble boosted tree) are presented in Table 1. The highest achieved accuracy is 98%, by MC-SVM. The other computed measures include the sensitivity rate of 98%, precision rate of 98.05%, F1-score of 98.025, and AUC of 0.99, while the computational time is 12.416 (seconds). The accuracy achieved on the DT classifier is 94.4%, and FNR is 5.6%, which is 3.6% higher than that of MC-SVM. This classifier’s computational time is 13.522 (seconds), which is higher than the time of MC-SVM. Similarly, the achieved accuracy on LDA, KNB, QSVM, F-KNN, cosine KNN, and EBT is 94.2%, 94.8%, 97.6%, 96.9%, 96.5%, and 96.3%, respectively. The FNR rate of each classifier is 5.8%, 5.2%, 2.4%, 3.1%, 3.5%, and 3.7%, respectively. Based on the accuracy and FNR, it is observed that the proposed method shows better results on MC-SVM. The computational time is also noted, and the minimum time is 12.115 (seconds) for F-KNN. However, this classifier’s accuracy is less than MC-SVM, and the time difference between both classifiers is minimal. Moreover, the scatter plots and confusion matrix are given for the verification of achieved accuracy for MC-SVM. The scatter plots are illustrated in Figure 6. Note that the scatter plot (left side) is original, and the scatter plot (right side) is predicted by the MC-SVM classifier. The confusion matrix of the classification results using MC-SVM is given in Figure 7. This shows that the correct prediction rate of COVID-19 is 97%.

**Table 1:** Proposed COVID-19-infected classification results for the selected imaging database. Best values are shown in bold.

Classifier	Evaluation Measures						
	Sensitivity (%)	Precision (%)	F1-Score (%)	AUC	Accuracy (%)	FNR (%)	Time (Seconds)
**MC-SVM**	**98.0**	**98.05**	**98.02**	**0.99**	**98.0**	**2.0**	12.416
DT	94.4	94.4	94.40	0.94	94.4	5.6	13.522
LDA	94.2	94.5	94.35	0.94	94.2	5.8	20.968
KNB	94.8	94.95	94.87	0.95	94.8	5.2	42.861
QSVM	97.6	97.65	97.62	**0.99**	97.6	2.4	15.202
F-KNN	96.9	95.45	96.17	0.97	96.9	3.1	**12.115**
Cosine KNN	96.5	96.5	96.50	0.99	96.5	3.5	12.334
EBT	96.3	96.35	96.32	0.97	96.3	3.7	20.253

We performed separate experiments to compare the proposed method results with previous steps (i.e., original features extraction and optimal deep features selection without fusion). These experiments support the performance of our proposed method. The results of original deep features are tabulated in Table 2, which shows the results calculated for both deep models (AlexNet and VGG16) for all selected classifiers. For AlexNet model features, MC-SVM attains the best accuracy of 94.4%, while the error rate and computational time are 5.6% and 39.366 (seconds), respectively. For VGG16, MC-SVM gives better results of 92.4%, while the error rate and computation time are 7.6% and 42.896 (seconds), respectively. It is noted that the performance of AlexNet is better in terms of accuracy and time. However, the accuracy of VGG16 is also near to the results of this model. The accuracy of other listed classifiers is also presented in this table.

For AlexNet features, the achieved accuracies are 88.3%, 90.1%, 91.6%, 92.3%, 90.7%, 91.1%, and 90.0%. Similarly, the computation time of each classifier is 43.266 (seconds), 53.042 (seconds), 86.116 (seconds), 45.125 (seconds), 36.846 (seconds), 42.200 (seconds), and 60.116 (seconds), respectively.

For VGG16 features, the achieved accuracy and computation time (seconds) for listed classifiers are (88.7%, 40.246), (89.6%, 59.160), (87.5%, 94.204), (92.4%, 44.116), (92.9%, 51.244), and (92.7%, 69.201), respectively. Based on these values, it is noted that the performance of AlexNet model features is better. Overall, the MC-SVM accuracy is better, but this accuracy is 4% less than the proposed technique accuracy. Moreover, the time consumption of each classifier is three times higher as compared with that in Table 1.

**Figure 8:**
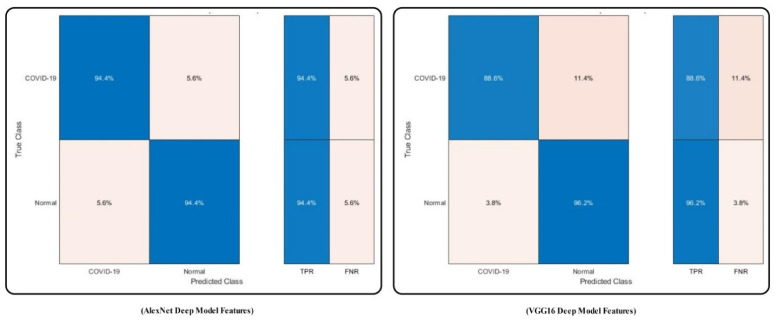
Confusion matrix of MC-SVM to verify the achieved accuracy using original deep model features after transfer learning.

The confusion matrix of MC-SVM using original AlexNet and VGG16 features is illustrated in Figure 8. The figure illustrates that the correct recognition rate of COVID19 is 94.4% and 88.6%, respectively.

The results of using the optimal deep features are tabulated in Table 3. MC-SVM achieved the highest accuracy of 96.2% and 94.2% for the AlexNet optimal and VGG16 optimal vectors, respectively. The error rate for each vector is 3.8% and 5.8%, respectively. Moreover, each vector’s computational time is 14.277 (seconds) and 15.004 (seconds), respectively. Compared with this accuracy, the error rate and computation time achieved with the original features of the deep model are as tabulated in Table 2, which shows that the accuracy of deep features is improved.

**Figure 9:**
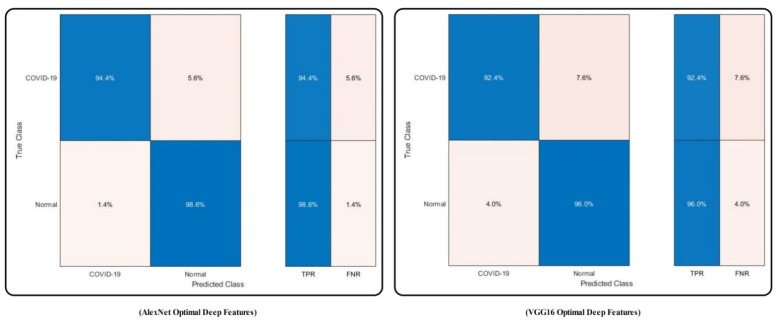
Confusion matrix of MC-SVM for the verification of the achieved accuracy using optimal deep features selection.

Moreover, the time is decreased by almost threefold. The confusion matrix of the results by MC-SVM for this experiment is also illustrated in Figure 9. Besides, the results for other classifiers are also presented in Table 3 and compared with Table 1. Note that the optimal deep features provide better performance. However, the individual deep vector’s accuracy is less than that of the proposed scheme, as tabulated in Table 1. The comparison between Table 1 and Table 3 shows that the accuracy of the proposed scheme is almost 2% better, and the time is nearly the same.

### 3.2. Analysis and Comparison

The performance of the proposed method with a combination of several features is analyzed in this section. This step’s primary aim is to support the proposed accuracy based on each involved step’s strength. As shown in Figure 1, the implanted method has four fundamental steps (i.e., contrast enhancement, deep learning features’ extraction, feature selection, and fusion). The results for each step are presented in Table 4. This table compares the effects of the proposed method with previous steps combinations. Initially, the AlexNet features are computed by employing contrast-enhanced images and an accuracy of 94.4%. In the next experiment, the AlexNet features are extracted without employing contrast-enhancing on images, achieving an accuracy of 91.7%. This step demonstrates that the utilization of contrast-enhanced images for AlexNet training improved the deep features.

Similarly, the experiments are performed on the VGG16 model with and without contrast-enhanced images and achieve an accuracy of 92.4% and 90.3%, respectively. The proposed optimal feature selection approach is later applied to both vectors and achieves accuracy of 96.2% and 94.2%, respectively. It shows that the accuracy is significantly increased after employing the optimal feature selection approach. Finally, the experiment is performed using the proposed scheme, and achieves an accuracy of 98%, which shows the strength of the proposed method.

**Figure 10:**
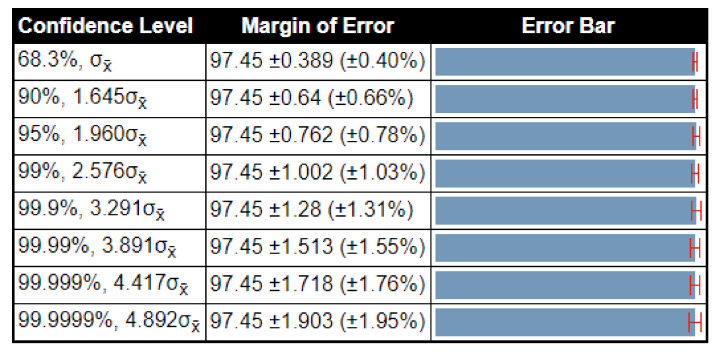
Confidence-interval-based analysis of the proposed method results.

The confidence interval based analysis is also conducted for the proposed method. The proposed method was executed 100 times and obtained a minimum accuracy of 96.9%, and the maximum accuracy is 98%. Through these values, the calculated standard deviation is 0.55, the variance is 0.3025, and the standard error mean (SEM) is 0.3889, respectively. Using these values, the confidence interval is plotted in Figure 10. Note that the margin of error (MOE) for the $95\%,\;1.960\sigma_{\bar{x}}$ confidence level is $97.45\pm 0.762\;\left(\pm 0.78\%\right)$, while the accuracy of the proposed method is almost consistent after several iterations.

**Figure 11:**
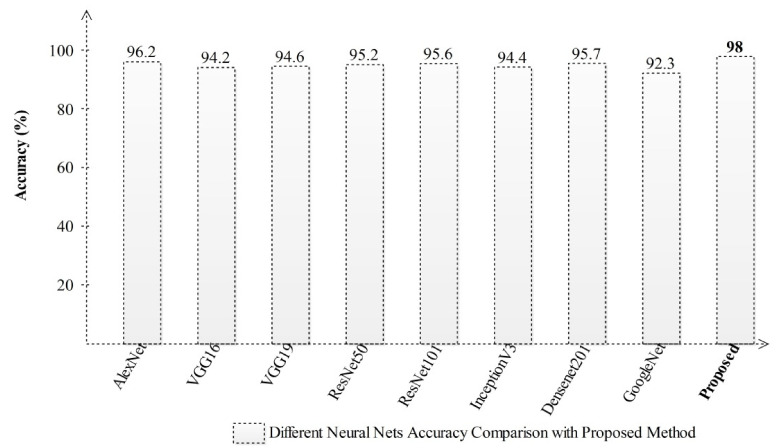
Comparison of the accuracy achieved with different deep learning models.

Besides a comparison with other neural network models, we have implemented several pre-trained models and performed experiments. The results are plotted in Figure 11, which shows that the proposed method outperforms other selected deep learning models.

**Figure 12:**
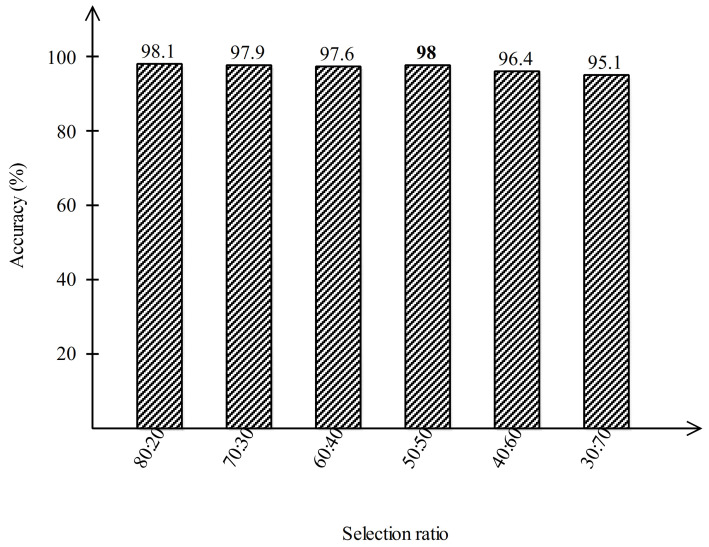
Classification results of MC-SVM for several training/testing ratios.

Moreover, the results are also computed on several training/testing ratios to justify the selection of the 50:50 ratio. Normally, researchers use the 70:30 ratio; however, for the fair process of training and testing, the 50:50 approach is much better. We calculated the results in several ratios, 80:20, 70:30, 60:40, 50:50, 40:60, and 30:70, and obtained more stable results for the ratio of 50:50. From Figure 12, it is clearly noted that the accuracy is degraded for ratios of 70:30, 60:40, 40:60, and 30:70. Hence, the accuracy achieved when using the 50:50 ratio was found to be much better.

Finally, the proposed method accuracy is compared with the existing techniques, presented in Table 5. In this table, 94.76% accuracy is achieved by [22]. They used the CT images having two classes, COVID-19 and normal, for classification purposes. The rest of the articles used the same CT images for the binary classification accuracy and achieved accuracy of 96.97% [59], 95.60% [60], and 95.1% [29]. The proposed method achieved an accuracy of 98%, which is improved compared with the existing techniques.

## 4. Conclusions

In this work, a new fully automated deep learning feature fusion-based method is proposed for the classification of chest CT images originating from COVID-19-infected and healthy subjects. In the proposed method, the first step is collecting a database from the Internet. The images in this database have low contrast; therefore, we implemented a new hybrid method. Through this method, the contrast was improved. This step plays a key role in the next step in obtaining useful characteristics. Fine tuning of two deep CNN models is performed according to the output classification classes. Transfer learning is employed on the modified fine-tuned models for training and deep features’ extraction. The extracted features of both layers included little redundant information, which misleads the classification process. Therefore, we proposed an entropy-controlled Firefly algorithm for the robust feature selection. The individual optimal features did not achieve the target accuracy; therefore, we employed new concatenation technique called parallel positive correlation. The final features are classified using MCSVM and achieved an accuracy of 98%. The number of redundant features, which still exist in this work, is the limitation of above-mentioned method.

The main limitation of the above technique is the number of redundant features, which still exist after feature selection and feature fusion. This problem can be considered in future studies. In future studies, we will also consider more patient data for the experimental validation process. The main limitation of the above technique is the number of redundant features, which still exist after feature selection and feature fusion. This problem can be considered in future studies.

In future studies, more datasets will be considered for the experimental process such as the COVID-CT dataset [59,61] and COVID-19 Pneumonia CT images dataset [62]. The COVID-19 Pneumonia CT dataset also includes a pneumonia class for classification purposes. Moreover, COVID-19 severity detection using deep learning-based segmentation will be considered as a future work as well [63,64,65,66,67,68].

## Figures and Tables

**Table 2:** Accuracy of deep models without using feature optimization and fusion steps.

Classifier	Deep Model Features		Evaluation Measures		
	AlexNet	VGG16	Accuracy (%)	Error Rate (%)	Time (Seconds)
**MC-SVM**	✓		**94.4**	**5.6**	39.366
		✓	**92.4**	**7.6**	42.896
DT	✓		88.3	11.7	43.266
		✓	88.7	11.3	**40.246**
LDA	✓		90.1	9.9	53.042
		✓	89.6	10.4	59.160
KNB	✓		91.6	8.4	86.116
		✓	87.5	12.5	94.204
QSVM	✓		92.3	7.7	45.125
		✓	93.6	6.4	49.334
F-KNN	✓		90.7	9.3	**36.846**
		✓	92.4	7.6	44.116
Cosine KNN	✓		91.1	8.9	42.200
		✓	92.9	7.1	51.244
EBT	✓		90.0	10.0	60.116
		✓	92.7	7.3	69.201

**Table 3:** Classification accuracy achieved using the optimal feature selection step.

Classifier	Optimal Deep Model Features		Evaluation Measures		
	AlexNet Optimal	VGG16 Optimal	Accuracy (%)	Error Rate (%)	Time (Seconds)
**MC-SVM**	✓		**96.2**	3.8	**14.277**
		✓	**94.2**	5.8	**15.004**
DT	✓		90.1	9.9	15.167
		✓	91.2	8.8	17.286
LDA	✓		92.4	7.6	23.004
		✓	91.6	8.4	24.120
KNB	✓		92.7	7.3	45.115
		✓	90.3	9.7	47.016
QSVM	✓		93.9	6.1	17.336
		✓	94.8	5.2	19.224
F-KNN	✓		92.6	7.4	15.296
		✓	93.5	6.5	16.110
Cosine KNN	✓		93.4	6.6	15.804
		✓	94.9	5.1	16.299
EBT	✓		92.8	7.2	23.134
		✓	94.1	5.9	23.896

**Table 4:** Comparison of the proposed accuracy with different feature combinations and steps.

Method	Accuracy (%)	Error Rate (%)
AlexNet + Contrast Enhancement Step	94.4	5.6
AlexNet without Contrast Step	91.7	8.3
VGG16 + Contrast Enhancement Step	92.4	7.6
VGG16 without Contrast Step	90.3	9.7
AlexNet + Contrast Step + Optimal Step	96.2	3.8
VGG16 + Contrast Step + Optimal Step	94.2	5.8
**Proposed Method**	**98.0**	**2.0**

**Table 5:** Comparison with existing techniques for COVID-19 classification.

Methods	Year	Accuracy (%)
[22]	2020	94.76
[33]	2020	96.97
[60]	2021	95.60
[29]	2021	95.1
**Proposed**		**98.0**

## Data Availability

The dataset is available from the Radiopaedia Website at https://radiopaedia.org/articles/covid-19-4?lang=gb (accessed on 23 September 2021).
